# What determines informal care need among community-dwelling older adults in China? Results from a longitudinal study

**DOI:** 10.1186/s12877-024-04843-3

**Published:** 2024-07-12

**Authors:** Liangwen Zhang, Shuyuan Shen, Wenzheng Zhang, Ya Fang

**Affiliations:** 1 State Key Laboratory of Molecular Vaccinology and Molecular Diagnostics, School of Public Health, Xiamen University, Xiamen, China; 2 Key Laboratory of Health Technology Assessment of Fujian Province, School of Public Health, Xiamen University, Xiang An Nan Road, Xiang An District, Xiamen, Fujian Province China

**Keywords:** Community home-based care, Care needs, Older adults, China

## Abstract

**Background:**

With an intensified aging population and an associated upsurge of informal care need in China, there is an ongoing discussion around what factors influence this need among older adults. Most existing studies are cross-sectional and do not focus on older people living in the community. Conversely, this study empirically explores the factors that affect informal care need of Chinese community-dwelling older individuals based on longitudinal data.

**Methods:**

This study constructed panel data using the China Health and Retirement Longitudinal Research Study (CHARLS) from 2011 to 2018 for analysis. Generalized linear mixed models were used to analyze the factors affecting reception of informal care, and linear mixed models were used to analyze the factors affecting informal care sources and intensity.

**Results:**

During the follow-up period, 7542, 6386, 5087, and 4052 older adults were included in 2011–2018, respectively. The proportion receiving informal care increased from 19.92 to 30.78%, and the proportion receiving high-intensity care increased from 6.42 to 8.42% during this period. Disability (*estimate* = 4.27, *P* < 0.001) and living arrangement (*estimate* = 0.42, *P* < 0.001) were the critical determinants of informal care need. The rural older adults reported a greater tendency to receive informal care (*estimate* = 0.14, *P* < 0.001). However, financial support from children did not affect informal care need (*P* > 0.05).

**Conclusions:**

At present, there is a great demand for the manpower and intensity of informal care, and the cost of informal care is on the rise. There are differences in informal care needs of special older groups, such as the oldest-old, living alone and severely disabled. In the future, the region should promote the balance of urban and rural care service resources, rationally tilt economic support resources to rural areas, reduce the inequality of long-term care resources, improve the informal care support system, and provide a strong community guarantee for the local aging of the older adults.

**Supplementary Information:**

The online version contains supplementary material available at 10.1186/s12877-024-04843-3.

## Background

Given the rapid growth of informal care need, which global population aging results in, it raises serious concerns about what factors influence this need and how to safeguard the sustainability of informal care system. Since 2000, China has become an aging society, progressing faster than low- and middle-income countries. According to the seventh national census in 2020, the population aged 60 years and older was up to 264 million, accounting for 18.7% of the total population in China. By 2030, the population aged 60 years and older is expected to reach 400 million, and the disabled population aged 65 years and older is estimated to reach 24.9 million [1, 2]. At the same time, informal care needs and costs for older individuals will increase significantly due to the inevitable population aging trends and highly rapid increase of the oldest-old [2].

Influenced by Confucianism’s filial culture, China relies on children and relatives to provide informal care [3]. Simultaneously, Chinese older adults tend to receive home-based care because informal care can improve their life satisfaction and quality of life [4]. Aging-in-place is to obtain continuous and comprehensive services in familiar family and community, which meets the psychological needs of the elderly and combines community-based care and home-based care. As well as the number of disabled older adults growing dramatically, there are a series of issues that make it hard for China to fully meet the care needs of older adults on multiple levels. These issues include family structure miniaturization, caregivers’ increasing financial burden, children’s continuing reduced ability to provide informal care, and a high risk of COVID-19 infection in nursing homes [5, 6]. Despite China’s substantial investment in developing nursing facilities, many beds remain unoccupied. Furthermore, nursing homes are frequently costly and distant from family, and many older adults fear losing face by transferring to a nursing home since this may imply that their offspring are not caring enough. Thus, China is experiencing challenges of imbalance between supply and need in informal care system.

Even though informal care is an integral part of long-term care, very little is known in the existing literature about factors impacting its receipt, sources and intensity in the Chinese older population. Most countries of the Organization for Economic Cooperation and Development (OECD) also emphasize the importance of community-based long-term care services and promote informal care to delay institutional care needs among older adults [7]. Therefore, it’s highly urgent for us to look into the determinants affecting informal care need and then provide evidence for the development of informal care support system.

### The importance and influencing factors of informal care

Informal care is the provision of care services by relatives, friends, neighbors, and volunteers to older people who have developed care requirements as a result of a partial or complete loss of self-care competence [8]. Confucianism, China’s primary value system, places a premium on family harmony and filial devotion. Informal care is provided to parents or spouses under this value system not only out of emotional dependence but also out of a strong sense of obligation to take on caregiving responsibilities as a family member, which has resulted in the predominance of children and spouses as informal care sources in China [9]. In terms of the factors, the location of an older adult’s living in urban or rural settings has a direct effect on their choice of care sources. Traditional values in rural areas have not changed as much as in urban areas where nuclear families are more common, so rural older people are more likely to receive informal care from their relatives than urban ones [10]. Moreover, Chinese children tend to provide financial support to their parents, which, according to Kemper’s theory [11], alters older adults’ economic status and then influences their preference for informal care sources. However, other scholars find that financial support has no effect on the informal care needs of Chinese older adults [12–14]. Additionally, women often bear more care responsibilities and face more enormous financial, physical, and emotional pressures than men, even though older women have greater care requirements than older men, leading to gender inequality in informal care [15–17]. Consequently, it is critical to improve supply capacity of care sources and refine informal care support system.

Informal care hours are used to associated with caregiver productivity and leisure time sacrifices, hence raising financial strain on families and lowering life quality. Angeles [18] found that providing temporary skilled nursing support to caregivers of older adults with dementia could reduce care hours and financial burden in OECD countries based on a systematic review of 307 studies. Wei Huang [19] predicted that informal care intensity for older adults with disabilities would increase annually from 2019 to 2028 in Xinjiang pastoral areas, with the highest cost of caregiving time and the fastest growth among older adults with mild disabilities. Hu [20] demonstrated that informal care sources were unequally distributed among older adults aged 80 years and older, and that oldest individuals who lived in urban areas or had with higher educational attainment received more care hours from their children. Furthermore, Hu [20] also highlighted that there were 27 million oldest adults in China and that every 1% increase in prediction accuracy could provide a more targeted allocation of care resources to 300,000 older adults. Altogether, figuring out what factors influence informal care intensity can enhance the accuracy of future prediction models. The development of prediction models can definitely promote personalized care service planning and efficient allocation of care resources.

The premise for perfecting informal care support system is to conduct a scientific and reasonable health economics evaluation of informal care, that is, to scientifically quantify the cost of care based on the assumption that informal care meets the quantity and quality of care needs of community-dwelling older adults. As key indicators of informal care needs, care sources and intensity can indirectly represent the cost of informal care, and determining their influencing factors is critical for the prediction of informal care cost. Accordingly, this study probes the influencing factors of informal care need among community-dwelling older adults in the context of Chinese characteristics, in order to provide a theoretical foundation for the improvement of informal care system. It also has implications for the construction of informal care systems in other countries.

### Current study on informal care needs

Theoretical and empirical evidence suggest that based on the Andersen’s model, predisposing, enabling, and need factors induced can affect informal care need among older adults. Chinese law and filial piety culture urge children to support their parents [21]. Meanwhile increased financial assistance will further significantly enhance the utilization of professional medical, healthcare, and nursing services among older adults [22]. However, the one-child policy and urbanization have resulted in the miniaturization of family structure, and then more children no longer dwell with older adults, making informal care less accessible. Little is known about whether the influence of these factors will change over time [14]. A study in Québec revealed that men are more inclined than women to prefer informal care, while individuals with lower income are less likely to favor formal care [23]. Also, there is an absence of research that focuses on community-dwelling older people and refines their informal care need [24]. Hence, this study examines the impact of predisposing, enabling, and need factors on the informal care need of Chinese community-dwelling older adults using the longitudinal data. We hypothesize that.

1. Among the predisposing factors, older adults who reside in the community, are of advanced age, female, living in rural areas, have lower levels of education, and are cohabiting or married are more likely to receive informal care compared to other types of older individuals. They require a greater number of informal care sources and higher intensity of informal care.
2. Community-dwelling older adults with higher income are less likely to receive informal care and require less informal care sources and lower informal care intensity.
3. Community-dwelling older adults whose children provide more financial support are less likely to receive informal care and require less informal care sources and lower informal care intensity.
4. Community-dwelling older adults with more chronic diseases, greater loneliness, poorer self-rated health, or severer disability tend to receive informal care and need more informal care sources and higher care intensity.

## Method

### Data and sample

Data come from the four follow-up data for older adults in China: the China Health and Retirement Longitudinal Research Study (CHARLS), a longitudinal survey of individuals aged 45 years and older [25]. The national baseline survey was conducted in 2011–2012, with wave 2 in 2013, wave 3 in 2015, and wave 4 in 2018, with multistage sampling and probability proportional to size sampling approaches. To ensure sample representativeness, the CHARLS baseline survey covered 150 countries/districts, 450 villages/urban communities, across the country, involving 17,708 individuals in 10,257 households, reflecting the mid-aged and older Chinese population collectively. The response rate for each wave was over 80%. We constructed panel data using the four waves of the CHARLS survey. Our sample was Chinese community-dwelling older adults aged 60 years and older in 2011, with those who chose to live in nursing homes, died, or were lost to follow-up in 2013–2018 excluded. Finally, 7542, 6386, 5087, and 4052 older adults were included in 2011–2018, respectively (Fig. S1).

### Measures

Informal care need is assessed using receipt of informal care, informal care sources, and informal care intensity [26]. Receipt of informal care was a dichotomous categorical variable comprised of the categories receiving informal care and not receiving informal care. There were five forms of care sources from which CHARLS respondents with a long-term disabled condition could choose, including care from a spouse, children, parents, other relatives, and the community, which resulted in many caregiving combinations. In this study, informal care sources referred to the number of care sources that older adults received, ranging from 0 to 5, and higher number of care sources indicated that older people need more human resource to provide assistance with ADL. Informal care intensity was measured by the number of care hours received in a week from their children, and more care hours indicated more caregiving time.

Age, education level, marital status, place of residence, economic status, internal and external care resource provision (informal family support network, provision of resources outside the home such as community-based care), and personal health status all influence older adults’ utilization of informal care services [27, 28]. As one of the most classic models for health service utilization research, Andersen’s model provides an excellent summary of inter-individual differences in the health service utilization behavior. This model is applicable to the study of long-term care, life quality of patients with chronic diseases, and health cost [29].

Under the guideline of Andersen’s model, in which an individual’s use of healthcare services and associated outcomes are viewed as a function of predisposing, enabling, and need factors, we selected independent variables. Firstly, sociodemographic traits and social structure were considered predisposing factors since they reflected an older individual’s sociocultural preferences and influence their informal care service utilization behavior. Secondly, enabling factors included the family resources available to an individual adult, which have an effect on the accessibility and availability of informal care services, which in this study referred to annual household income and regular financial support provided by children. Most researchers agree that the higher older adults’ objective purchasing power, the larger their ability to transform potential need into actual need, and the lesser their proclivity for informal care. Thirdly, the need factors were measured by objective health status and subjective health status, affecting the likelihood of seeking and utilizing informal care. The objective health status was subdivided into two components: the prevalence of chronic diseases and the independence in ADL function. The functional disability basic ADLs was divided into four health statues: healthy (0 ADL), mild disability (1–2 ADL), moderate disability (3–4 ADL), and severe disability (5–6 ADL), using the Katz Index where six ADLs were bathing, dressing, grooming, transferring, eating, and toileting [30]. The subjective health status was measured by the self-perceived loneliness and the self-perceived health based on two CHARLS entries: “I feel lonely” and “How do you feel about your health”.

### Data analytic procedure

STATA version 15.1 was used for data cleaning and R version 4.1.2 was used for data analyses. Predisposing factors, enabling factors, need factors, receipt of informal care, and the most common combinations of informal care sources and the different degrees of informal care intensity in 2011–2018 were shown in Table 1. Besides, bivariate analyses were conducted to examine group differences (gender and place of residence) across all variables at the baseline (Table S1).

The Generalized Linear Mixed Models (GLMMs, models 1–2) was applied to analyze receipt of informal care among selected participants. The Linear Mixed Models (LMMs, models 3–6) was used to perform informal sources and intensity among selected participants. Both GLMMs and LMMs are designed to analyze longitudinal data due to their merits in adjusting the random effects from repeated measures on the same subject, and the within-subject and between-subject variability. During the modeling, GLMMs and LMMs could capture the effect of both time-invariant factors (e.g., gender) and the time-variant factors (e.g., residence type). The CHARLS survey timepoints (2011, 2013, 2015, 2018) was also included in the data analysis to model change in informal care need. In addition to exploring what factors determine informal care needs, we were interested in the effects of gender, residence, and financial support from children on estimates of informal care needs over time. Therefore, this study evaluated the interaction terms between these three independent variables and the survey timepoint in the GLMMs (model 2) and LMMs (model 4,6), so as to model the effects of the three primary variables on the change in informal care needs among community-dwelling older adults between 2011 and 2018 in China.

The Akaike Information Criterion (AIC) was compared to estimate the model fit, and a lower number indicates a better model fit. In addition, the full model was tested among each sub-group based on gender (female and male), and place of residence (urban and rural) to explore the group-specific associations. For the mixed models, the sampling probabilities varied exogenously by design. In this study, both the weighted and unweighted coefficients were consistent, but the weighted results tended to be less precise (e.g. larger standard errors). Therefore, it only reported the unweighted results in this paper.

## Results

### Descriptive statistics

At present, Chinese community-dwelling older adults have a growing need for informal care in terms of sources and intensity, while informal care costs are increasing. As shown in Table 1, and 30.58% of community-dwelling older adults received informal care, 28.64% chose informal care provided by their spouse or children, and 8.42% required high intensity care hours (30 + h/week) in 2018. It was worth noting that 90.4% of older adults required regular financial support from their children, and financial support in this study did not include hidden costs incurred by children providing informal care due to database limitations, such as medical subsidies, reduced wage income, nutrition costs, and transportation costs associated with caring for the elderly, demonstrating that informal care costs should not be underestimated [31].

Rural-urban migration is a significant characteristic of China. In the trend toward growing urbanization and urban-rural mobility, older adults in rural areas have a lower degree of economic development and retirement security than those in urban areas. According to Table 1, the proportion of community-dwelling older people in rural regions reached 82.18% in 2018, a considerable increase from the proportion in 2011, and in brief the rural retirement situation is not positive. Between 2011 and 2018, the proportion of oldest adults aged 80 years and older climbed from 8.6 to 14.16%, while the proportion of elderly people with two or more chronic conditions increased from 45.86 to 68.21%. Although the proportion of older adults in healthy status has remained around 90%, only 18.29% of all have self-perceived good health status, and the proportion of older adults receiving informal care increased from 19.92 to 30.78%, indicating that older adults, even if they live alone, have informal care needs, including life care and mental health needs.

**Table 1:** Descriptive statistics of community-dwelling older adults

	Community-dwelling older adults				
	2011 (*N* = 7542) n (%)	2013 (*N* = 6386) n (%)	2015 (*N* = 5087) n (%)		2018 (*N* = 4052) n (%)
**Age**					
60 to 69 years old	4669 (61.91)	3511 (54.98)	2503 (49.21)	1239 (30.58)	
70 to 79 years old	2224(29.49)	2187 (34.25)	2068 (40.65)	2239 (55.26)	
80 years and older	649(8.60)	688 (10.77)	516 (10.14)	574 (14.16)	
**Gender**					
Male	3778 (50.09)	3182 (49.83)	2551 (50.15)	1991 (49.14)	
Female	3764 (49.91)	3204 (50.17)	2536 (49.85)	2061 (50.86)	
**Place of residence**					
Urban	2977 (39.47)	2352 (36.83)	1088 (21.39)	722 (17.82)	
Rural	4565 (60.53)	4034 (63.17)	3999 (78.61)	3330 (82.18)	
**Education**					
Below primary school	4333 (57.45)	3718 (58.22)	2909 (57.19)	2321 (57.28)	
Primary/middle school	2676 (35.48)	2248 (35.20)	1858 (36.52)	1489 (36.75)	
High school and above	533 (7.07)	420 (6.58)	320 (6.29)	242 (5.97)	
**Marital status**					
Unmarried/Divorced	1605 (21.32)	1479 (23.16)	1214 (23.86)	1103 (27.22)	
Married	5934 (78.68)	4907 (76.84)	3873 (76.14)	2949 (72.78)	
**Living arrangement**					
Alone	1921 (25.48)	1667 (26.10)	1336 (26.26)	1186 (29.27)	
Not alone (with children/a spouse/other relatives)	5621 (74.52)	4719 (74.90)	3751 (73.74)	2866 (70.73)	
**Yearly income**					
None	4011 (53.18)	1001 (15.67)	323 (6.35)	378 (9.33)	
¥1 to ¥19,999	2809 (37.25)	4661 (72.99)	4087 (80.34)	3618 (89.29)	
¥20,000 and more	722 (9.57)	724 (11.34)	677 (13.31)	56 (1.38)	
**Monthly financial support from children**					
None	3565 (47.27)	901 (14.11)	655 (12.88)	389 (9.60)	
¥1 to ¥999	3826 (50.73)	5080 (79.55)	3820 (75.09)	3235 (79.84)	
¥1,000 and more	151 (2.00)	405 (6.34)	612 (12.03)	428 (10.56)	
**Chronic conditions**					
None	1903 (25.23)	1627 (25.48)	869 (17.08)	445 (10.98)	
1 condition	2181 (28.92)	1859 (29.11)	1364 (26.81)	843 (20.81)	
2 conditions and more	3458 (45.85)	2900 (45.41)	2854 (56.11)	2764 (68.21)	
**Self-perceived loneliness**					
Not lonely	6009 (79.67)	5391 (84.42)	4009 (78.81)	3078 (75.96)	
Lonely	1533 (20.33)	995 (15.58)	1078 (21.19)	974 (24.04)	
**Self-perceived health**					
Good	1476 (19.57)	1304 (20.42)	963 (18.93)	741 (18.29)	
Fair	3315 (43.95)	2975 (46.59)	2487 (48.89)	1895 (46.77)	
Bad	2751 (36.48)	2107 (32.99)	1637 (32.18)	1416 (34.94)	
**Disability**					
Independent	6773 (89.81)	5707 (89.37)	4553 (89.50)	3616 (89.24)	
Mild disability	550 (7.29)	481 (7.53)	425 (8.35)	353 (8.71)	
Moderate disability	117 (1.55)	115 (1.80)	73 (1.44)	53 (1.31)	
Severe disability	102 (1.35)	83 (1.30)	36 (0.71)	30 (0.74)	
**Receipt of informal care**					
No	6040 (80.08)	4347 (68.07)	3526 (69.31)	2813 (69.42)	
Yes	1052 (19.92)	2039 (31.93)	1561 (30.69)	1239 (30.58)	
**Types of informal care sources**					
Care from a spouse only	782 (10.37)	807 (12.64)	662 (13.01)	491 (12.12)	
Care from children only	534 (7.08)	699 (10.95)	568 (11.17)	457 (11.28)	
Care from a spouse and children	103 (1.37)	354 (5.54)	221 (4.34)	177 (4.37)	
Care from others	83 (1.10)	179 (2.80)	110 (2.17)	113 (2.81)	
No informal care	6040 (80.08)	4347 (68.07)	3526 (69.31)	2813 (69.42)	
**Degrees of informal care intensity**					
No informal care	6040 (80.08)	4347 (82.49)	3526 (69.31)	2813 (69.42)	
Fewer than 10 h weekly	525 (6.96)	1368 (7.00)	670 (13.17)	586 (14.46)	
10 to 30 h weekly	493 (6.54)	320 (5.01)	461 (9.06)	312 (7.70)	
More than 30 h weekly	484 (6.42)	351 (5.50)	430 (8.46)	341 (8.42)	

### Generalized linear mixed models and linear mixed models

**Fig. 1:**
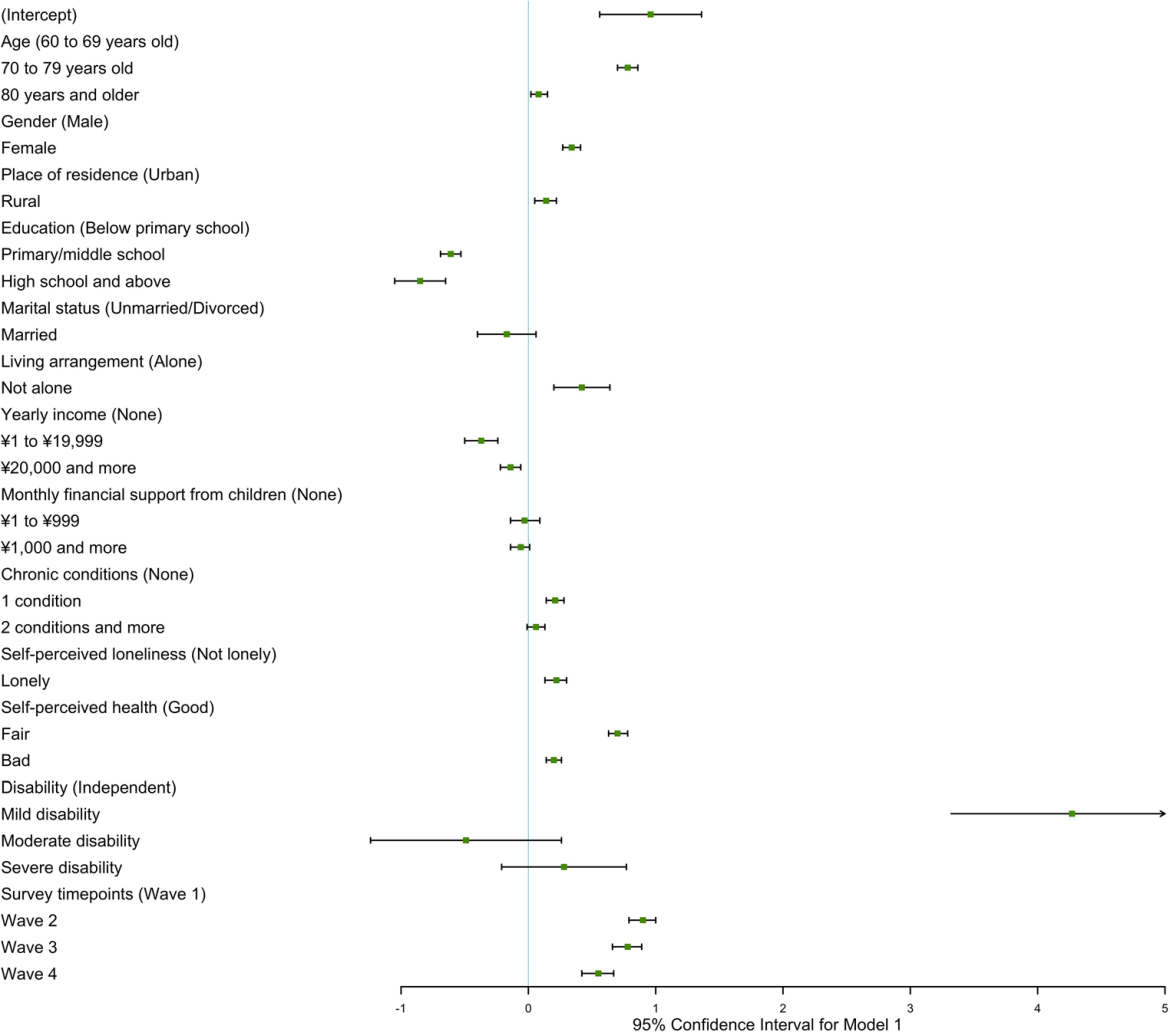
Generalized linear mixed model for receipt of informal care without interaction

**Fig. 2:**
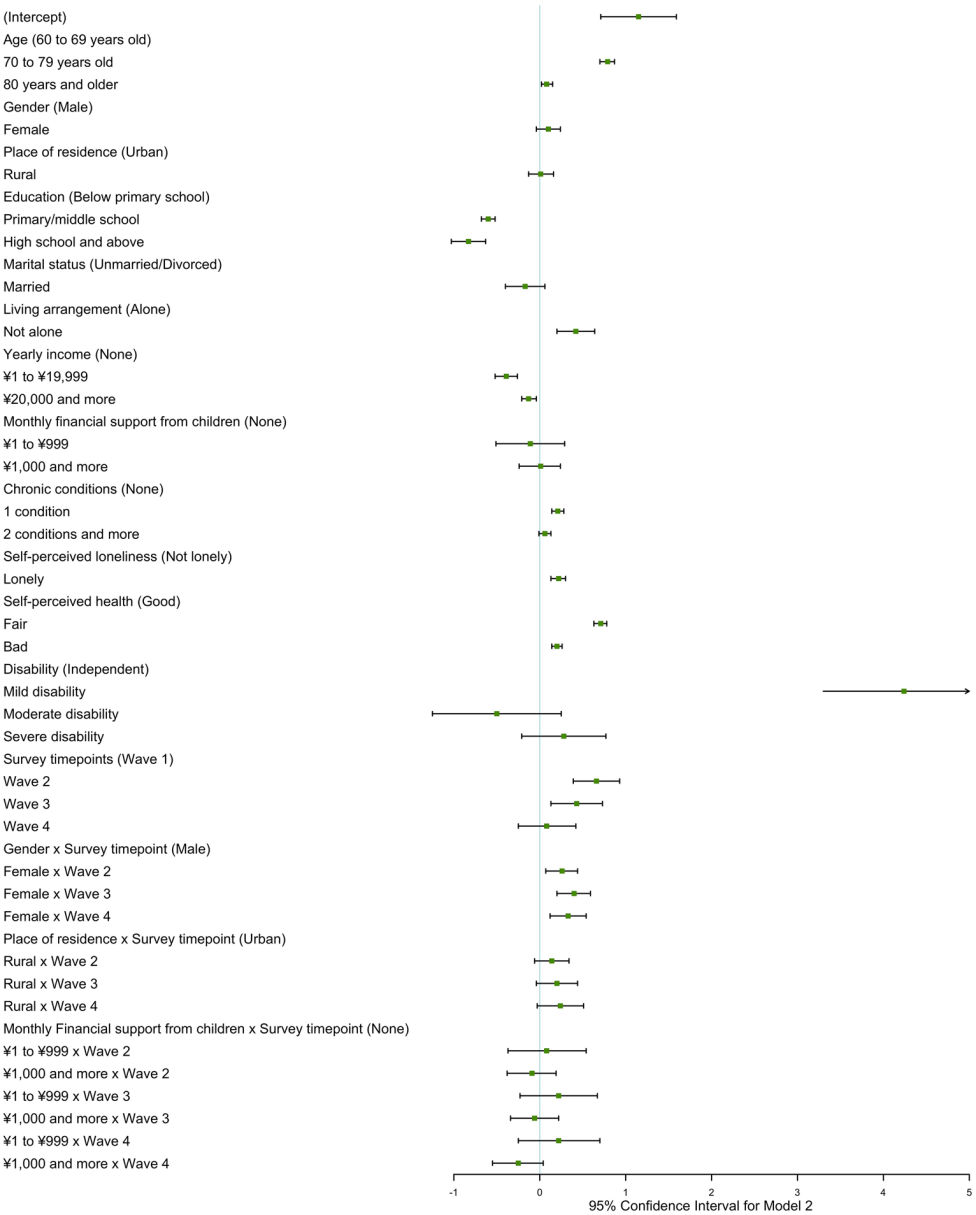
Generalized linear mixed model for receipt of informal care with interaction

Figures 1 and 2 show that age, gender, place of residence, education, living arrangement, household income, chronic conditions, loneliness, self-perceived health, and disability are associated with receipt of informal care among older individuals living in the community. Compared to the young age group (60 to 69 years old), the middle age group (70 to 79 years old) and the old age group (80 years and older) were more likely to receive informal care and had more care sources and higher care intensity, due to deteriorating physical condition in the aging process (Table 2). Older women reported more care resources and more care hours than older men (*estimate* = 0.08, *P* < 0.001 for care sources, and *estimate* = 1.34, *P* < 0.001 for care intensity). Based on the fixed effect for gender and its interaction effects with the survey timepoints, older women reported more care sources than older men during 2011–2018 (Table 2). Also, it found that rural participants got more care sources than urban participants in the longitudinal study. In addition, all models indicated that a greater level of education was associated with a decreased propensity for older adults to receive informal care, as well as a reduced need for care resources and shorter care hours. It was supported that older people living with others got more care sources than those living alone (Figs. S1 and S2). However, it did not find marital status could influence informal care need of older adults in full models.

**Table 2:** Linear mixed model for informal care sources and intensity among community-dwelling older adults in 2011–2018

Characteristics	Informal care sources		Informal care intensity	
	Model 3 Estimate [95% CI]	Model 4 Estimate [95% CI]	Model 5 Estimate [95% CI]	Model 6 Estimate [95% CI]
**(Intercept)**	0.66 *** [0.44, 0.88]	0.71 [−0.49, 0.93]	49.97 *** [44.72, 55.23]	51.78 [44.94, 55.61]
**Predisposing factors**				
**Age (60 to 69 years old)**				
70 to 79 years old	0.13 *** [0.12, 0.15]	0.13 *** [0.12, 0.15]	9.67 *** [8.24, 11.10]	9.72 *** [8.29, 11.15]
80 years and older	0.02 *** [0.01, 0.03]	0.02 *** [0.01, 0.04]	3.02 *** [1.94, 4.10]	3.02 *** [1.94, 4.10]
**Gender (male)**				
Female	0.08 *** [0.04, 0.07]	0.01 [−0.02, 0.02]	1.34 * [1.11, 2.55]	−1.22 [−3.23, −0.79]
**Place of residence (urban)**				
Rural	0.02 ** [0.01, 0.03]	−0.01 [−0.04, 0.01]	−1.24 [−2.59, 0.11]	−1.92 [−4.02, 0.17]
**Education (below primary school)**				
Primary/middle school	−0.11 *** [−0.12, −0.09]	−0.10 *** [−0.12, −0.09]	−2.00 ** [−3.30, −1.71]	−1.95 ** [−2.24, −1.65]
High school and above	−0.12 *** [−0.14, −0.09]	−0.12 *** [−0.15, −0.09]	−2.97 * [−5.60, −1.35]	−2.93 * [−4.56, −1.29]
**Marital status (unmarried/divorced)**				
Married	0.02 [−0.02, 0.06]	0.02 [−0.01, 0.06]	−2.38 [−5.68, 0.93]	−2.45 [−5.75, 0.85]
**Living arrangement (alone)**				
Not alone (with children/a spouse/other relatives)	0.07 *** [0.03, 0.10]	0.07 *** [0.03, 0.10]	2.88 [−0.24, 6.00]	2.93 [−0.19, 6.05]
**Enabling factors**				
**Yearly income (none)**				
¥1 to ¥19,999	−0.04 *** [−0.06, −0.02]	−0.05 *** [−0.07, −0.03]	−1.51 [−3.28, 0.25]	−1.50 [−3.28, 0.29]
¥20,000 and more	−0.01 [−0.03, 0.01]	−0.01 [−0.02, 0.01]	−0.07 [−1.24, 1.10]	0.27 [−0.93, 1.46]
**Monthly financial support from children (none)**				
¥1 to ¥999	−0.01 [−0.03, 0.01]	−0.01 [−0.06, 0.05]	0.56 [−1.22, 2.35]	0.23 [−4.80, 5.25]
¥1,000 and more	−0.01 [−0.02, 0.01]	0.01 [−0.02, 0.04]	0.75 [−0.41, 1.90]	1.73 [−1.40, 4.87]
**Need factors**				
**Chronic conditions (none)**				
1 condition	0.03 *** [0.02, 0.04]	0.03 *** [0.02, 0.04]	1.51 ** [1.41, 1.60]	1.50 ** [0.40, 2.59]
2 conditions and more	0.01 [−0.01, 0.02]	0.01 [−0.01, 0.02]	0.27 [−0.78, 1.33]	0.27 [−0.79, 1.33]
**Self−perceived loneliness (not lonely)**				
Lonely	0.04 *** [0.13, 0.30]	0.04 *** [0.02, 0.06]	0.09 [−1.39, 1.57]	0.10 [−1.38, 1.57]
**Self−perceived health (good)**				
Fair	0.12 *** [0.02, 0.06]	0.12 *** [0.11, 0.14]	5.06 *** [3.84, 6.28]	5.10 *** [3.88, 6.32]
Bad	0.05 *** [0.03, 0.06]	0.05 *** [0.04, 0.06]	1.85 *** [1.14, 2.79]	1.87 *** [1.12, 2.62]
**Disability (Independent)**				
Mild disability	0.61 *** [0.56, 0.65]	0.67 *** [0.56, 0.65]	68.60 *** [64.77, 72.43]	68.52 *** [64.74, 72.39]
Moderate disability	−0.28 *** [−0.32, −0.24]	−0.28 *** [−0.32, −0.24]	7.32 *** [3.68, 10.97]	7.27 *** [3.62, 10.92]
Severe disability	0.09 *** [0.05, 0.13]	0.09 *** [0.05, 0.13]	−0.22 [−3.73, 3.28]	−0.35 [−3.85, 3.16]
**Survey timepoints (Wave 1)**				
Wave 2	0.18 [−0.14, 0.49]	0.10 [−0.21, 0.40]	5.15 [−1.33, 11.63]	3.75 [−0.26, 7.75]
Wave 3	0.14 [−0.18, 0.45]	0.04 [−0.27, 0.34]	6.31 [−0.22, 12.84]	−0.54 [−4.94, 3.85]
Wave 4	0.11 [−0.21, 0.42]	0.01 [−0.30, 0.32]	2.87 [−3.70, 9.43]	1.10 [−3.84, 6.04]
**Gender × survey timepoint (male)**				
Female × Wave 2		0.08 *** [0.05, 0.11]		2.58 [−0.30, 5.47]
Female × Wave 3		0.09 *** [0.06, 0.13]		6.20 *** [3.11, 9.28]
Female × Wave 4		0.08 *** [0.04, 0.12]		2.81 [−0.51, 6.12]
**Place of residence × survey timepoint (urban)**				
Rural × Wave 2		0.05 ** [0.02, 0.08]		−0.25 [−3.27, −2.78]
Rural × Wave 3		0.07 *** [0.03, 0.11]		4.16 * [1.14, 7.17]
Rural × Wave 4		0.05 * [0.01, 0.10]		0.29 [−3.80, 4.37]
**Monthly financial support from children × survey timepoint (none)**				
¥1 to ¥999 × Wave 2		−0.01 [−0.08, 0.06]		0.02 [−6.13, 6.18]
¥1,000 and more × Wave 2		−0.03 [−0.08, 0.01]		−1.55 [−5.43, 2.34]
¥1 to ¥999 × Wave 3		0.02 [−0.05, 0.09]		2.29 [−3.76, 8.34]
¥1,000 and more × Wave 3		−0.02 [−0.07, 0.02]		−2.36 [−6.23, 1.51]
¥1 to ¥999 × Wave 4		0.03 [−0.04, 0.11]		2.24 [−4.27, 8.76]
¥1,000 and more × Wave 4		−0.06* [−0.11, −0.01]		−1.62* [−5.77, 2.53]
AIC	32343.15	32266.67	239249.5	239208.5

Note: *P < 0.05; *P* < 0.01;*P* < 0.001. Reference group is listed in (--)

Compared to older adults without income, those who had higher income were less likely to receive informal care (¥1 to ¥19,999: *estimate*=−0.37, *P* < 0.001;¥20,000 and more: *estimate*=−0.14, *P* < 0.001). Although, there was no evidence to support the interaction between financial support from children and the survey timepoints. Moreover, need factors are predictive of informal care needs during 2011–2018, for example, where it is observed that self-rated fair health status (*estimate* = 5.10, *P* < 0.001) and self-rated bad health status (*estimate* = 1.87, *P* < 0.05) are associated with higher levels of informal care intensity. Refer to the supplementary figures for detailed results.

## Discussion

Our findings further show that the factors influencing informal care needs for community-dwelling older adults in China based on CHARLS data from 2011 to 2018. The study demonstrated an increasing trend of informal care needs among community-dwelling older adults as they age and experience more functional disabilities. This trend holds implications for long-term care policies internationally, as populations globally are experiencing rapid population aging. Other countries should monitor changing dynamics of elderly care needs over time. Place of residence (urban vs. rural) and economic status were found to influence older adults’ choices of informal care sources. A balanced consideration of diverse regional and income-level needs is warranted when allocating care resources across populations. Family structural changes have weakened children’s capacity for elderly care. Strengthening community support is vital to compensate for vulnerabilities in family caregiving systems, an issue exacerbated by ongoing transformation of family structures worldwide. Gender emerged as an impact factor, with women tending to have greater care requirements while primarily shouldering care responsibilities, potentially leading to caregiver burden. Other nations must also address gender inequities in elderly care provision and outcomes. Sustaining an equitable and efficient informal care support system necessitates a holistic perspective accounting for multiple influencing factors. This holds lessons for developing sustainable elderly care models to meet basic living needs while alleviating family caregiver strain, crucial objectives for aging societies globally.

For *Hypothesis 1*, our findings support that age, gender, place of residence, education, and living arrangement were associated with informal care need among older adults. Among them, those who are older, female, live in rural areas, have higher education levels, and live alone have higher care resource and intensity needs. Interestingly, the association between marital status and informal care need was not established. Aging is associated with a loss of muscle mass, leading to frailty and functional disability, making older adults require daily life support [33]. In the Chinese context, women tend to assume caregiving roles and take on more physical and mental stress [14]. During the pandemic, it has been reported that female informal caregivers are more negatively affected than male informal caregivers, as indicated by higher levels of anxiety and lower quality of life [34]. It is unequal that older women have higher levels of informal care needs and experience a more extended period of end-of-life disability than older men, but they have higher caregiving burdens and more caregiving responsibilities than older men [35–37]. In rural areas, community-based care facilities are poorly developed, the number of nursing homes is low, and their cost is beyond the affordability of rural households. Moreover, older adults have less access to informal care when their children live farther away [38]. Thus, place of residence and living arrangements determine the availability of informal care. Older adults with lower educational attainment tend to have lower abilities to pay and are more likely to choose lower-cost informal care when informal care is needed [14].

This study also supports *Hypothesis 2*, that community-dwelling older adults with lower income were more likely to receive informal care, although the effect of income on informal care intensity was not supported. Higher-income level has been negatively associated with the receipt of informal care among older adults [39, 40]. However, *Hypothesis 3* was not supported, that financial support from children affects informal care needs. In China, informal care cost is usually underestimated, and the hidden financial loss of children is always ignored, because it is considered children’s duty to support their parents [31, 32, 41].

Our findings also support *Hypothesis 4*, that need factors were the primary force behind informal care need, including chronic conditions, self-perceived loneliness, self-perceived health, and disability. It is shown that older persons with a poorer health status are more likely to receive informal care with greater care resources and greater care intensity, which is consistent with the findings of other researchers [3, 26, 42].

The findings of this study contribute to a growing body of research identifying factors that determine receipt, sources, and intensity of informal care. This longitudinal study demonstrates that different dimensions of informal care need are affected by different factors. This study points to the need for innovative community-based programs that establish daycare centers, give daily life support to older people, and teach children professional nursing skills, especially older women and female informal caregivers [8]. What is more, there are urban-rural differences in both the supply and need of informal care due to the dual socioeconomic structure of the urban-rural divide [43]. Policymakers should consider the differences in income, perceptions and service provision between urban and rural older people when developing policies for improving informal care support systems, such as constructing community-based service facilities, developing teams of nursing professionals, and expanding long-term care insurance coverage. Additionally, informal care costs need to be taken seriously, with uniform quantitative evaluation indicators and financial subsidies in different proportions according to the actual situation of urban and rural areas and special populations [31, 44]. It is recommended that the subsidies include both the elderly and informal caregivers. This aims to improve the material conditions of older people and stimulate informal caregivers to take care of older people, thus alleviating the pressure of caregiving on families and maintaining the sustainability of informal care system.

The identified factors associated with informal care needs among community-dwelling older individuals provide valuable insights into the potential determinants of informal care requirements in different cultural and social-economic contexts. Understanding these factors can help inform policies and interventions aimed at supporting informal caregivers and meeting the care needs of older adults in various countries, especially in developing countries. Furthermore, recognizing the vital role of informal caregivers in the healthcare system is crucial across nations. Informal caregivers play a significant role in providing essential care and support to older adults. Their contribution to the health services industry should be acknowledged and supported through appropriate policies, resources, and recognition of their valuable work. By understanding the dynamics of informal care, countries can work towards creating sustainable and comprehensive support systems that enhance the health and quality of life of older adults while recognizing and supporting the invaluable role of informal caregivers.

## Conclusions

This study revealed that the factors including age, gender, place of residence, education, living arrangement, household income, chronic conditions, loneliness, self-perceived health, and disability are associated with informal care need among community-dwelling older individuals in China from 2011 to 2018. Older adults who are female, the oldest, living rural, living alone, having lower income, feeling lonely, and severely disabled require more informal care need. Informal caregivers are the backbone of the healthcare system to the extent that their contribution to health services industry is essential. Further study is needed to forecast the trajectory of informal care need among community-dwelling older adults, as well as development and implement of informal care support system aimed at fostering better health and well-being.

Of course, this study still has certain limitations. Firstly, we were limited by the availability of sample size, and therefore, we were unable to address the issue of regional heterogeneity. Secondly, there might be some potential influencing factors that we did not include. Thirdly, since the data is not first-hand information, the selection bias and other potential random errors in sample selection weaken our inference to a certain extent. Lastly, due to the self-report nature of the questionnaire collection, there may also be some biases in the data collection process.

### Electronic supplementary material

Below is the link to the electronic supplementary material.

Supplementary Material 1

## Data Availability

The datasets analyzed during the current study are publicly available in the CHARLS repository at http://charls.pku.edu.cn/en.
