# Supplementary material for: What determines informal care need among community-dwelling older adults in China? Results from a longitudinal study
Source: BMC Geriatr. 2024 Jul 12;24:597. doi: 10.1186/s12877-024-04843-3 (PMC11241955; doi:10.1186/s12877-024-04843-3)
Supplement: Supplementary file 1 — Supplementary Material 1 [file 12877_2024_4843_MOESM1_ESM.docx]

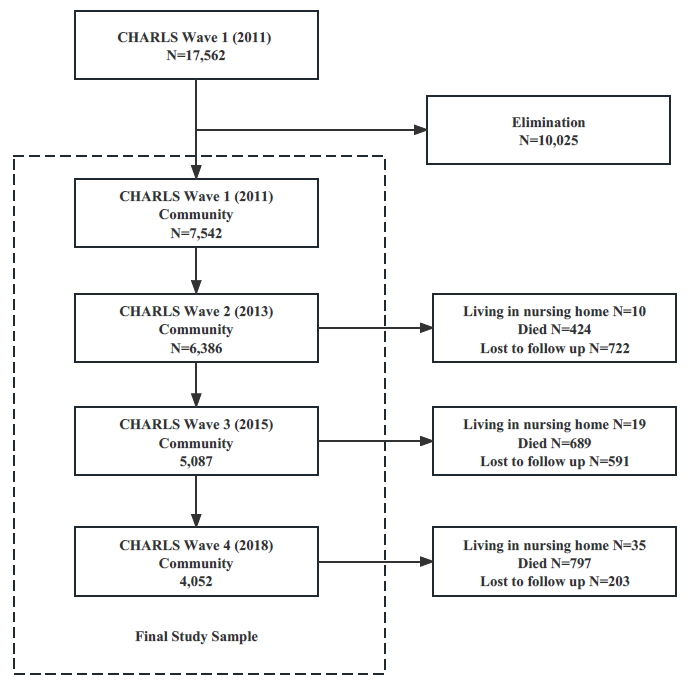


**Figure S1 Final study sample selection flow chart**

Note: Elimation included being under 60 years of age, opting for formal care, and the number of missing entries reaching 80% of the total number of entries.

**Table S1 Background** **characteristics according to gender and place of residence at the baseline survey**

|  | **Gender** | | | **Place of residence** | | |
| --- | --- | --- | --- | --- | --- | --- |
| **Characteristics** | **Male** | **Female** | **χ ^2^(df)/**  **t-test (df)** | **Urban** | **Rural** | **χ ^2^(df)/**  **t-test (df)** |
| **Predisposing factors** |  |  |  |  |  |  |
| **Age** |  |  | 13.27 (2)** |  |  | 9.28 (2)** |
| 60 to 69 years old | 31.30 | 30.60 |  | 23.65 | 38.25 |  |
| 70 to 79 years old | 15.06 | 14.43 |  | 12.40 | 17.09 |  |
| 80 years and older | 3.73 | 4.88 |  | 3.42 | 5.19 |  |
| **Gender** | -- | -- | -- |  |  | 5.50 (1)* |
| Male |  |  |  | 19.11 | 30.99 |  |
| Female |  |  |  | 20.36 | 29.54 |  |
| **Place of residence** |  |  | 5.50 (1)* | -- | -- | -- |
| Urban | 19.11 | 20.36 |  |  |  |  |
| Rural | 30.99 | 29.54 |  |  |  |  |
| **Education** |  |  | 670.87 (2)*** |  |  | 592.74 (2)*** |
| Below primary school | 21.44 | 36.01 |  | 17.43 | 40.02 |  |
| Primary/middle school | 23.55 | 11.93 |  | 16.27 | 19.21 |  |
| High school and above | 5.11 | 1.96 |  | 5.77 | 1.30 |  |
| **Marital status** |  |  | 321.72 (1)*** |  |  | 1.35 (1) |
| Unmarried/Divorced | 6.44 | 14.88 |  | 8.14 | 13.18 |  |
| Married | 43.65 | 35.03 |  | 31.33 | 47.35 |  |
| **Living arrangement** |  |  | 217.14 (1)*** |  |  | 5.85 (1)* |
| Alone | 9.06 | 16.41 |  | 9.45 | 16.02 |  |
| Not alone (with children/a spouse/other relatives) | 41.04 | 33.49 |  | 30.02 | 44.51 |  |
| **Enabling factors** |  |  |  |  |  |  |
| **Yearly Income** |  |  | 252.44 (2)*** |  |  | 810.77 (2)*** |
| None | 23.57 | 29.61 |  | 15.26 | 37.92 |  |
| ¥1 to ¥19,999 | 19.23 | 18.02 |  | 16.18 | 21.07 |  |
| ¥20,000 and more | 7.29 | 2.28 |  | 8.03 | 1.54 |  |
| **Monthly Financial support from children** |  |  | 14.23 (2)*** |  |  | 171.86 (2)*** |
| None | 24.65 | 22.62 |  | 21.82 | 25.44 |  |
| ¥1 to ¥999 | 24.34 | 26.39 |  | 16.46 | 34.28 |  |
| ¥1,000 and more | 1.10 | 0.90 |  | 1.19 | 0.81 |  |
| **Need factors** |  |  |  |  |  |  |
| **Chronic Conditions** |  |  | 18.25 (2)*** |  |  | 16.29 (2)*** |
| None | 13.60 | 11.63 |  | 9.51 | 15.72 |  |
| 1 condition | 14.56 | 14.36 |  | 10.74 | 18.18 |  |
| 2 conditions and more | 21.93 | 21.92 |  | 19.23 | 26.62 |  |
| **Self-perceived loneliness** |  |  | 65.50 (1)*** |  |  | 74.67 (1)*** |
| Not lonely | 41.79 | 37.88 |  | 33.41 | 46.26 |  |
| Lonely | 8.30 | 12.03 |  | 6.06 | 14.27 |  |
| **Self-perceived health** |  |  | 52.37 (2)*** |  |  | 110.68 (2)*** |
| Good | 10.50 | 9.07 |  | 8.82 | 10.75 |  |
| Fair | 23.32 | 20.63 |  | 19.09 | 24.86 |  |
| Bad | 16.27 | 20.21 |  | 11.56 | 24.92 |  |
| **Disability** |  |  | 9.89 (3)* |  |  | 8.18 (3)* |
| Independent | 45.52 | 44.29 |  | 35.65 | 54.15 |  |
| Mild disability | 3.22 | 4.07 |  | 2.63 | 4.67 |  |
| Moderate disability | 0.70 | 0.85 |  | 0.53 | 1.02 |  |
| Severe disability | 0.65 | 0.70 |  | 0.66 | 0.69 |  |
| **Baseline survey (wave 1)** |  |  |  |  |  |  |
| **Receipt of informal care** |  |  | 35.89 (1)*** |  |  | 16.27 (1)*** |
| No | 41.50 | 38.59 |  | 32.52 | 47.56 |  |
| Yes | 8.59 | 11.32 |  | 6.95 | 12.97 |  |
| **Informal care sources** |  |  | -5.85 (7443.7)*** |  |  | -4.05 (6724)*** |
| **Informal care intensity** |  |  | -1.84 (7428.40) |  |  | 1.55 (5820) |
| Note: **P<0.05*; ***P<0.01*; ****P<0.001*. | | | | | | |

**Table S2 Generalized linear mixed model for receipt of informal care among community-dwelling older adults in 2011-2018**

|  | **Receipt of informal care** | |
| --- | --- | --- |
| **Characteristics** | **Model 1**  Estimate [95% CI] | **Model 2**  Estimate [95% CI] |
| **(Intercept)** | 0.96 *** [0.56, 1.36] | 1.15 *** [1.01, 1.59] |
| **Predisposing factors** |  |  |
| **Age (60 to 69 years old)** |  |  |
| 70 to 79 years old | 0.78 *** [0.69, 0.86] | 0.79 *** [0.70, 0.87] |
| 80 years and older | 0.08 ** [0.02, 0.14] | 0.08 ** [0.02, 0.15] |
| **Gender (Male)** |  |  |
| Female | 0.34 *** [0.27, 0.41] | 0.10 [-0.04, 0.24] |
| **Place of residence (Urban)** |  |  |
| Rural | 0.14 ** [0.05, 0.22] | 0.01 [-0.13, 0.16] |
| **Education (Below primary school)** |  |  |
| Primary/middle school | -0.60 *** [-0.68, -0.52] | -0.60 *** [-0.68, -0.52] |
| High school and above | -0.85 *** [-1.04, -0.53] | -0.83 *** [-0.99, -0.63] |
| **Marital status (Unmarried/Divorced)** |  |  |
| Married | -0.17 [-0.39, 0.06] | -0.17 [-0.40, 0.05] |
| **Living arrangement (Alone)** |  |  |
| Not alone (with children/a spouse/other relatives) | 0.42 *** [0.20, 0.64] | 0.42 *** [0.20, 0.64] |
| **Enabling factors** |  |  |
| **Yearly income (None)** |  |  |
| ¥1 to ¥19,999 | -0.37 *** [-0.50, -0.24] | -0.39 *** [-0.52, -0.26] |
| ¥20,000 and more | -0.14 *** [-0.22, -0.05] | -0.13 ** [-0.21, -0.04] |
| **Monthly financial support from children (None)** |  |  |
| ¥1 to ¥999 | -0.03 [-0.14, 0.09] | -0.11 [-0.51, 0.29] |
| ¥1,000 and more | -0.07 [-0.14, 0.01] | -0.01 [-0.24, 0.24] |
| **Need factors** |  |  |
| **Chronic conditions (None)** |  |  |
| 1 condition | 0.21 *** [0.14, 0.28] | 0.21 *** [0.14, 0.29] |
| 2 conditions and more | 0.06 [-0.01, 0.12] | 0.06 [-0.01, 0.13] |
| **Self-perceived loneliness (Not lonely)** |  |  |
| Lonely | 0.22 *** [0.13, 0.30] | 0.22 *** [0.13, 0.30] |
| **Self-perceived health (Good)** |  |  |
| Fair | 0.70 *** [0.62, 0.78] | 0.71 *** [0.63, 0.78] |
| Bad | 0.20 *** [0.14, 0.26] | 0.20 *** [0.14, 0.26] |
| **Disability (Independent)** |  |  |
| Mild disability | 4.27 *** [3.32, 5.21] | 4.25 *** [3.30, 5.19] |
| Moderate disability | -0.49 [-1.23, 0.26] | -0.50 [-1.25, 0.25] |
| Severe disability | 0.28 [-0.21, 0.77] | 0.28 [-0.21, 0.77] |
| **Survey timepoints (Wave 1)** |  |  |
| Wave 2 | 0.90 *** [0.79, 0.99] | 0.66 *** [0.39, 0.93] |
| Wave 3 | 0.78 *** [0.66, 0.89] | 0.43 ** [0.13, 0.73] |
| Wave 4 | 0.55 *** [0.42, 0.67] | 0.08 [-0.25, 0.42] |
| **Gender x Survey timepoint (****Male)** |  |  |
| Female x Wave 2 |  | 0.26 ** [0.07, 0.44] |
| Female x Wave 3 |  | 0.40 *** [0.20, 0.60] |
| Female x Wave 4 |  | 0.33 ** [0.12, 0.54] |
| **Place of residence x Survey timepoint (Urban)** |  |  |
| Rural x Wave 2 |  | 0.14 [-0.06, 0.34] |
| Rural x Wave 3 |  | 0.20 [-0.04, 0.44] |
| Rural x Wave 4 |  | 0.24 [-0.03, 0.51] |
| **Monthly Financial support from children x Survey timepoint (None)** |  |  |
| ¥1 to ¥999 x Wave 2 |  | 0.08 [-0.37, 0.54] |
| ¥1,000 and more x Wave 2 |  | -0.09 [-0.38, 0.19] |
| ¥1 to ¥999 x Wave 3 |  | 0.22 [-0.23, 0.67] |
| ¥1,000 and more x Wave 3 |  | -0.06 [-0.34, 0.22] |
| ¥1 to ¥999 x Wave 4 |  | 0.22 [-0.25, 0.70] |
| ¥1,000 and more x Wave 4 |  | -0.25 [-0.55, 0.04] |
| **AIC** | 20501.1 | 20490.2 |

Note: **P<0.05*; ***P<0.01*; ****P<0.001*. Reference group is listed in (--).


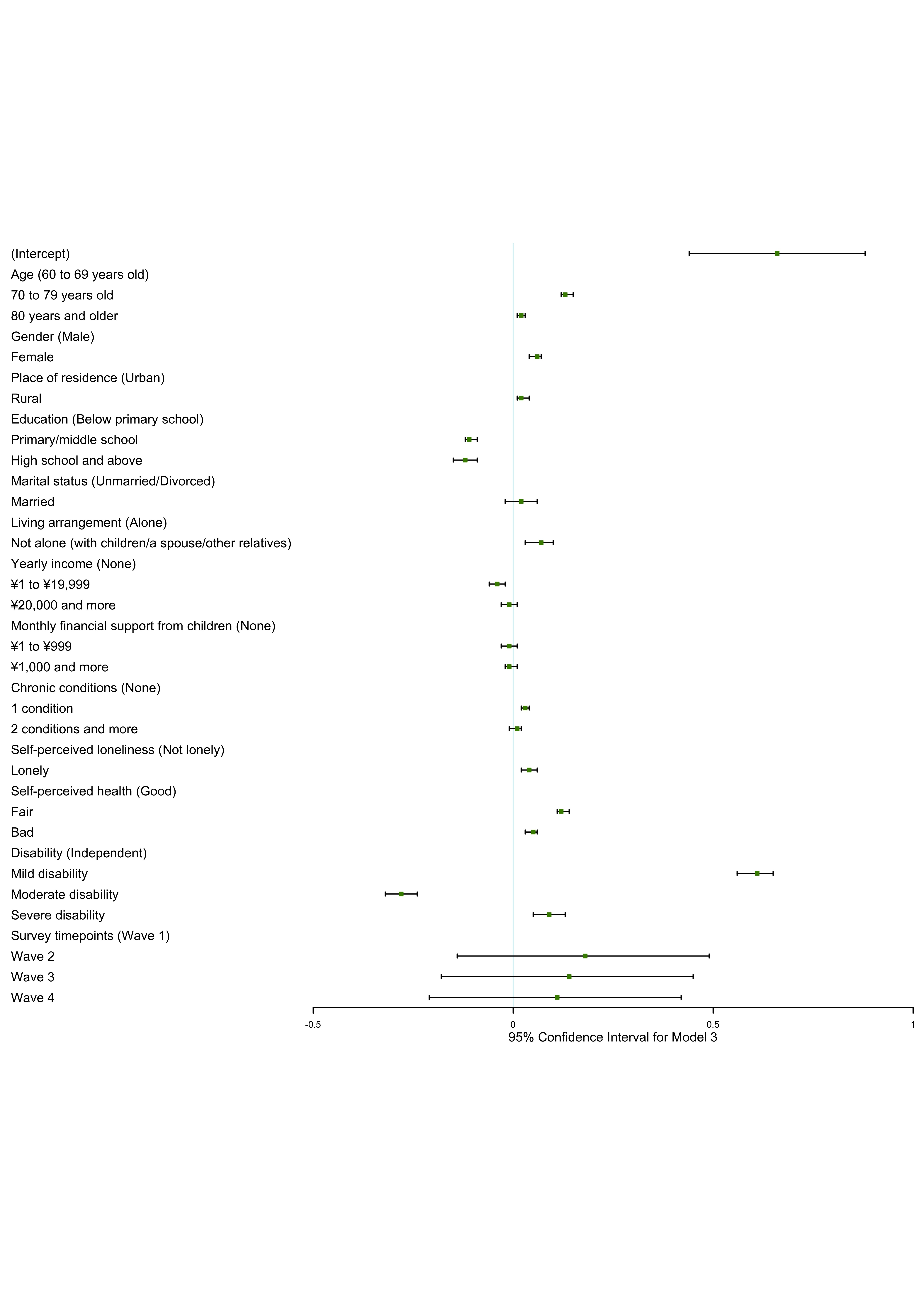


**Figure S2 Linear mixed model for informal care sources without interaction**


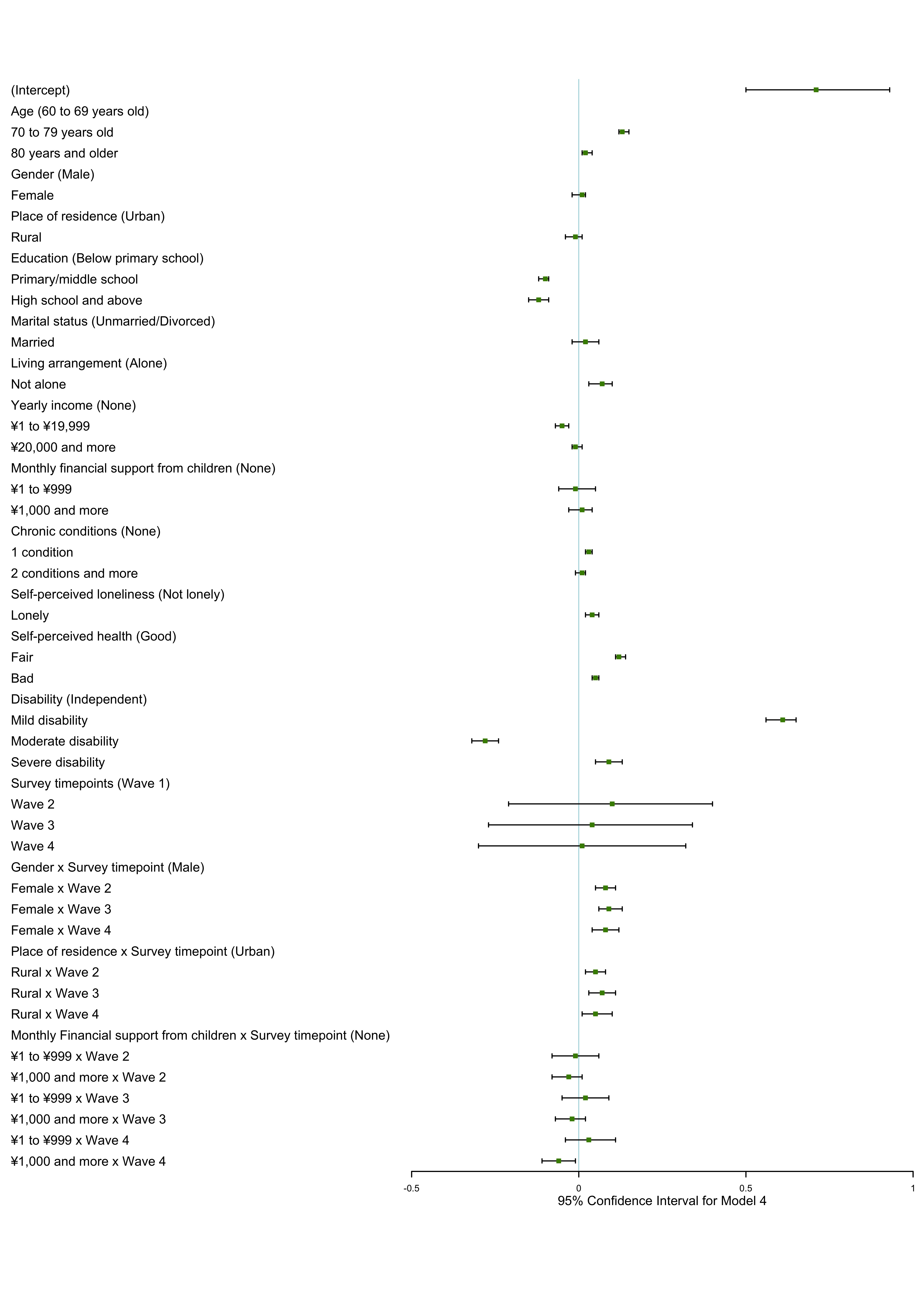


**Figure S3 Linear mixed model for informal care sources with interaction**


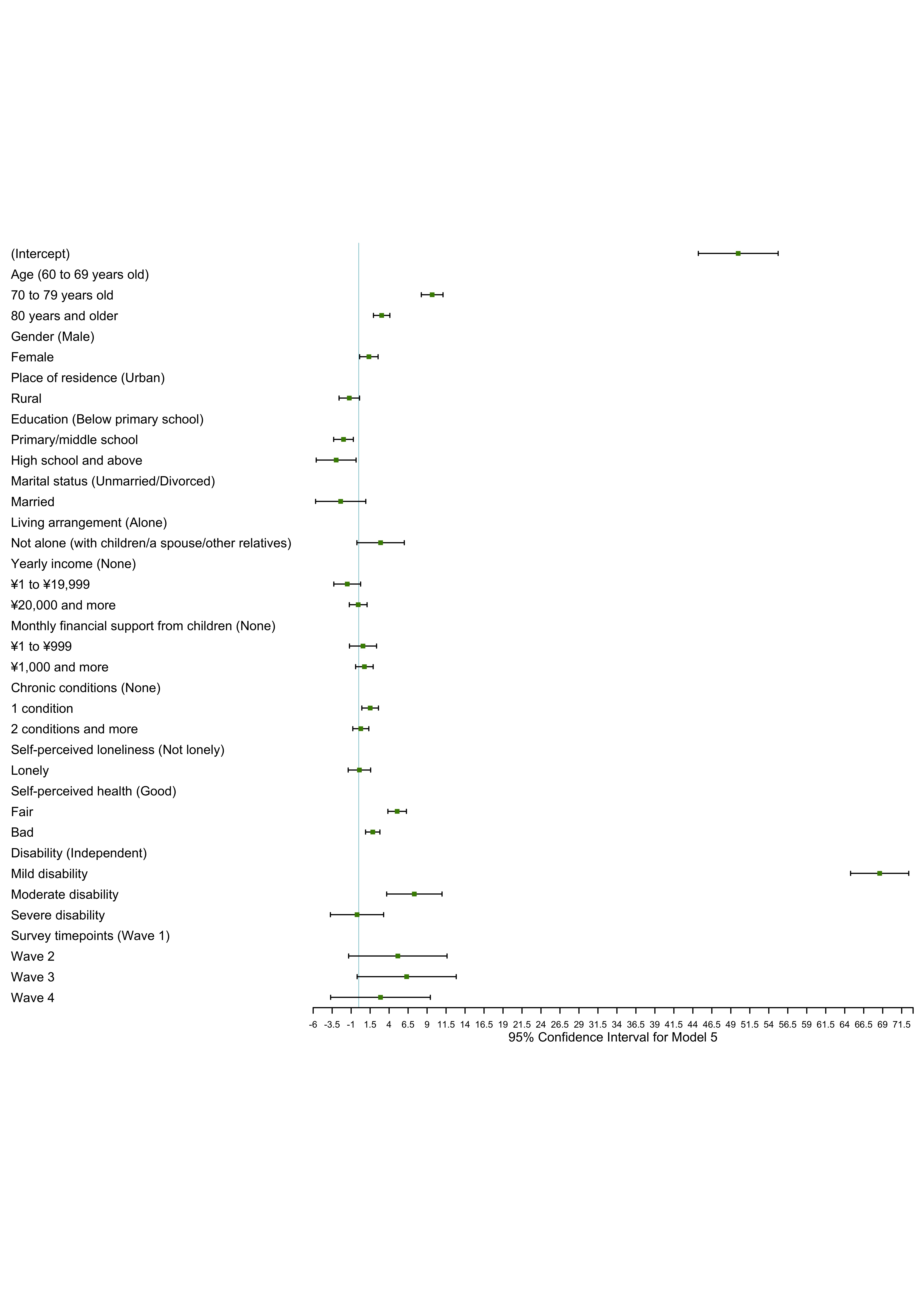


**FigureS4 Linear mixed model for informal care intensity without interaction**


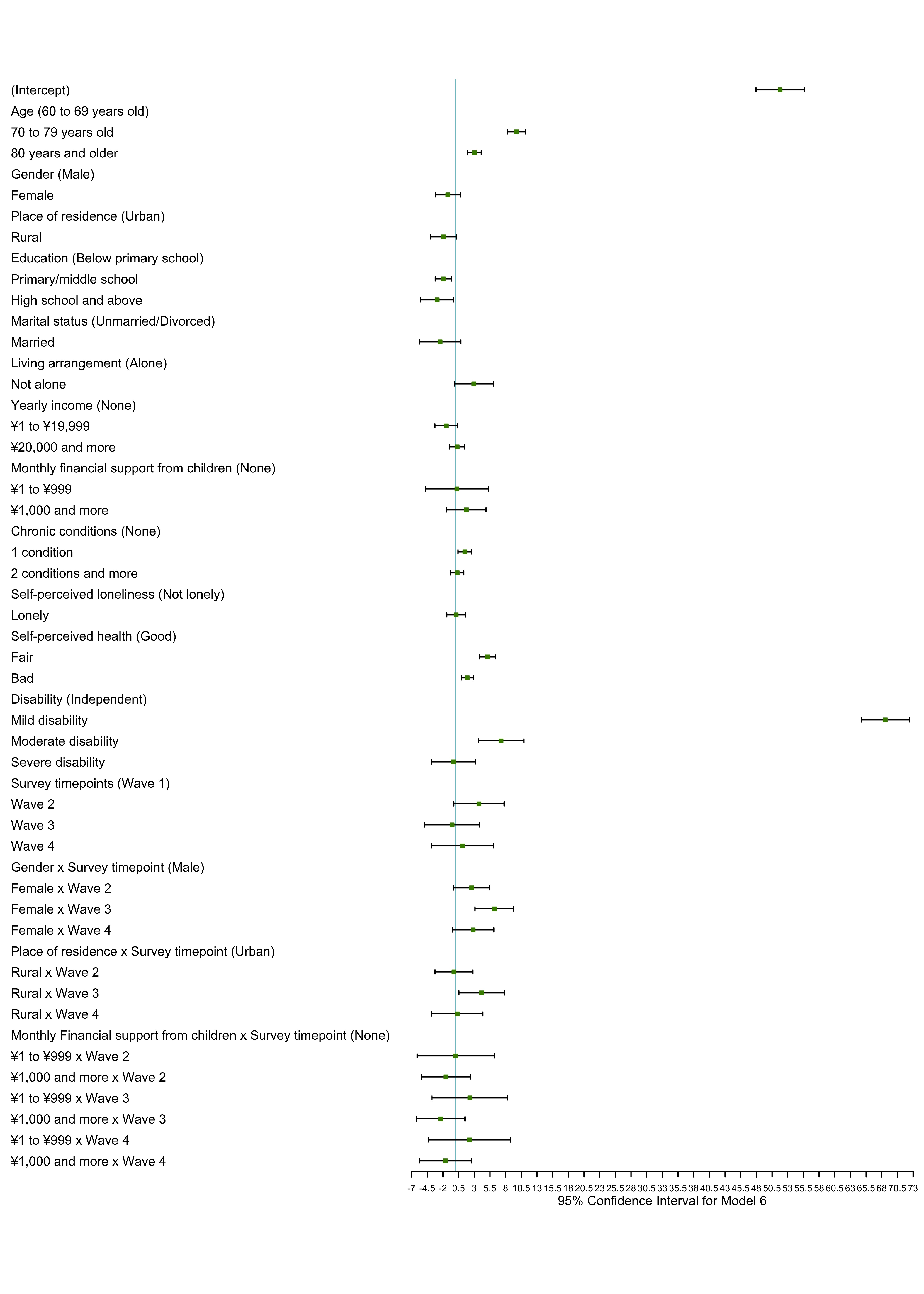


**Figure S5 Linear mixed model for informal care intensity with interaction**
